# Joint Modeling of Longitudinal Markers and Time-to-Event Outcomes: An Application and Tutorial in Patients After Surgical Repair of Transposition of the Great Arteries

**DOI:** 10.1161/CIRCOUTCOMES.120.007593

**Published:** 2021-10-22

**Authors:** Sara J. Baart, Roel L.F. van der Palen, Hein Putter, Roula Tsonaka, Nico A. Blom, Dimitris Rizopoulos, Nan van Geloven

**Affiliations:** Department of Biostatistics (S.J.B., D.R.), Erasmus MC, Rotterdam, the Netherlands.; Department of Epidemiology (S.J.B., D.R.), Erasmus MC, Rotterdam, the Netherlands.; Division of Pediatric Cardiology, Department of Pediatrics (R.L.F.v.d.P., N.A.B.), Leiden University Medical Center, the Netherlands.; Department of Biomedical Data Sciences, Section Medical Statistics (H.P., R.T., N.v.G.), Leiden University Medical Center, the Netherlands.; Division of Pediatric Cardiology, Department of Pediatrics, Amsterdam UMC, University of Amsterdam, the Netherlands (N.A.B.).

**Keywords:** aortic valve, body surface area, echocardiography, heart defects, congenital, heart disease, longitudinal studies, prognosis, survival analysis

## Abstract

Most patients with congenital heart disease survive into adulthood; however, residual abnormalities remain and management of the patients is life-long and personalized. Patients with surgical repair of transposition of the great arteries, for example, face the risk to develop neoaortic valve regurgitation. Cardiologists update the prognosis of the patient intuitively with updated information of the cardiovascular status of the patient, for instance from echocardiographic imaging.

**Methods:**

Usually a time-dependent version of the Cox model is used to analyze repeated measurements with a time-to-event outcome. New statistical methods have been developed with multiple advantages, of which the most prominent one being the joint model for longitudinal and time-to-event outcome. In this tutorial, the joint modeling framework is introduced and applied to patients with transposition of the great arteries after surgery with a long-term follow-up, where repeated echocardiographic values of the neoaortic root are evaluated against the risk of neoaortic valve regurgitation.

**Results:**

The data are analyzed with the time-dependent Cox model as benchmark method, and the results are compared with a joint model, leading to different conclusions. The flexibility of the joint model is shown by adding the growth rate of the neoaortic root to the model and adding repeated values of body surface area to obtain a multimarker model. Lastly, it is demonstrated how the joint model can be used to obtain personalized dynamic predictions of the event.

**Conclusions:**

The joint model for longitudinal and time-to-event data is an attractive method to analyze data in follow-up studies with repeated measurements. Benefits of the method include using the estimated natural trajectory of the longitudinal outcome, great flexibility through multiple extensions, and dynamic individualized predictions.

## Abstract 2

Supplemental Digital Content is available in the text.

The management of patients with congenital heart disease is a personalized life-long follow-up in which, during outpatient visits, the current cardiac status and the change in cardiac status are assessed. Survival into adulthood is expected for almost all congenital heart diseases,^1^ but residual abnormalities are often present and may progress over time. Cardiologists intuitively adjust their assessment of prognosis based on the change in clinical status and additional imaging parameters.

Large datasets with risk factors and outcomes of patients make it possible to quantify who will and who will not likely experience adverse events or disease progression. To evaluate the association between a longitudinal marker, that is, a measurement repeatedly monitored over time, such as cardiac dimension, and the occurrence of an event over time, such as valve leakage, several statistical methods can be used. A traditional approach is to use a Cox regression with a time-dependent covariate. However, in recent years, new methods have been developed that offer several advantages over the time-dependent Cox model. The most prominent method is called joint modeling, referring to the simultaneous modeling of the longitudinal marker and the time-to-event outcome.

Because this new technique is not well known to clinical researchers, the aim of this tutorial is to provide an introduction to the joint modeling framework. The basic concepts are explained, and advantages of the joint model (JM) are illustrated by applying the method to patients after surgical repair of transposition of the great arteries (TGA). These patients face the risk of developing neoaortic root (Root) dilatation and neoaortic valve regurgitation (AR) during follow-up postsurgery. In the study used as an illustration, longitudinal echocardiographic follow-up data, such as Root dimensions, are evaluated against the risk of developing AR.

We exhibit the different analysis steps that are needed for an accurate analysis. A time-dependent Cox model will be used as a benchmark method. Then we introduce the JM for longitudinal and time-to-event data methods and illustrate differences between the 2 methods. Several extensions of the JM are employed to demonstrate the flexibility of the framework. Lastly, the JM is used to obtain dynamic predictions of the risk of the event for individual patients. This is an important step to make the models available for use in clinical practice, so when a patient comes into the clinic for a new visit, their risk can be updated using the new measurements.

## Data

The data come from a study of retrospective assessment of serial echocardiographic data from patients born with a TGA, who were treated by arterial switch operation (ASO) shortly after birth with a maximum 35 years follow-up (1977–2015). Patients with TGA with the following morphological subtypes were included: TGA and intact ventricular septum, TGA with ventricular septal defect, and patients with double outlet right ventricle with subpulmonary ventricular septal defect (Taussig-Bing anomaly). Echocardiographic data from all patients were analyzed by 2 observers by grading AR and measuring Root dimensions as previously described.^2^ In short, neoaortic diameters were determined from 2-dimensional parasternal long-axis view at different neoaortic levels; neoaortic valve annulus, Root, and neoaortic sinotubular junction. AR was graded semiquantitatively on a 4-point scale: grade 1, nontrivial; grade 2, mild; grade 3, moderate; grade 4, severe regurgitation. Only good quality image data were assessed at approximately the following intervals after ASO: at 3, 6, 9, and 12 months, at 2, 3, and 5 years and thereafter with 5-year intervals including the last available follow-up or image data before reoperation for Root pathology. Demographic data and data on morphological and surgical details were retrospectively assessed from hospital and outpatient records. Surgical details included for this study consisted of anatomy of the pulmonary valve (bicuspid or tricuspid valve leaflet); pulmonary artery banding for left ventricular training before ASO; ASO performed > 6 months of age. For this tutorial, data from the neoaortic valve status, Root diameter, the body surface area (BSA), surgical details, and sex were used for the analysis and illustration.

The data set consists of 345 patients with a total of 1223 repeated measurements. On average, patients had 3 to 4 repeated measurements. The median age at operation was 9 days after birth (interquartile range, 5–17 days). The median age at first assessment was 1.9 (interquartile range, 0.3–7.1) years and at the last follow-up 9.8 (interquartile range, 4.4–15.3) years. At follow-up visits, the neoaortic dimensions are measured as well as the neoaortic valve status. During follow-up, 111 patients reached the end point of AR ≥grade 2 (32%). The median age at which this end point was assessed was 8.5 [interquartile range, 3.5–11.5] years. Other important clinical variables can be found in Table 1.

**Table 1.**
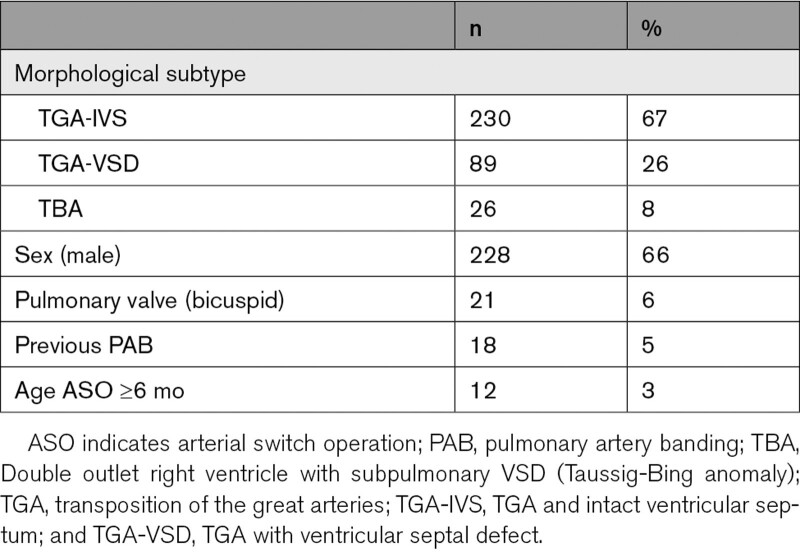
Baseline Characteristics

### Data Visualization

**Figure 1.**
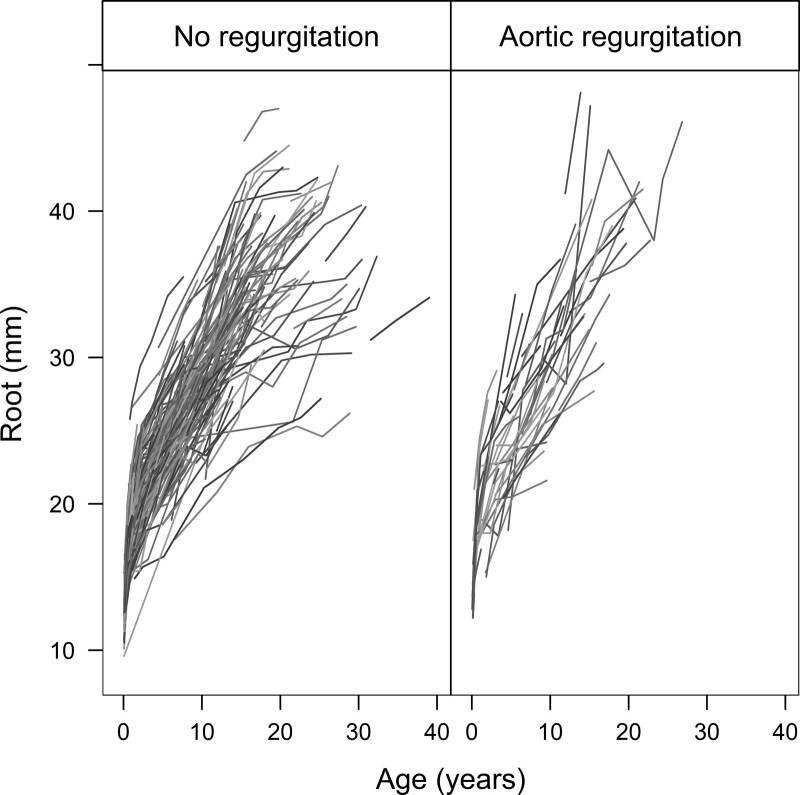
**Individual trajectories of the neoaortic root dimension.** Each line represents the trajectory of the echocardiographic measurements of the neoaortic root diameter of time in years. The lines in the **left** represent the patients that do not develop neoaortic valve regurgitation ≥grade 2, and the **right** represents the patients that do.

A first step into the analyses is a visual exploration of the marker trajectories over time. In this tutorial, we will be investigating the trajectories of the Root diameter (mm). Figure 1 shows the evolutions of this marker, where each line represents the evolution of a patient. The *x* axis represents the age of the patients in years. The figure is split into patients that reached the event of interest (AR≥grade 2) and the patients without the event.

All patients demonstrate rapid growth at the beginning of the follow-up period as children, which progresses but attenuates from adulthood. There are, however, apparent differences in the evolutions; some patients’ Root dimension grows faster than others. Moreover, not every patient had the same number of measurements, the same start of follow-up, or the same follow-up time depending on the availability of high-quality echocardiograms and the year of operation, respectively. Outpatient visits were planned according to the patients’ clinical status and residual abnormalities on the echocardiogram.

### Modeling Strategies

Fictive simulated data, similar to the real dataset, is publically available online at https://github.com/SaraBaart/Joint-Model-Tutorial. In the Data Supplement and on the GitHub page code is provided to run the analyses discussed in this tutorial.

### Time-Dependent Cox Model

**Figure 2.**
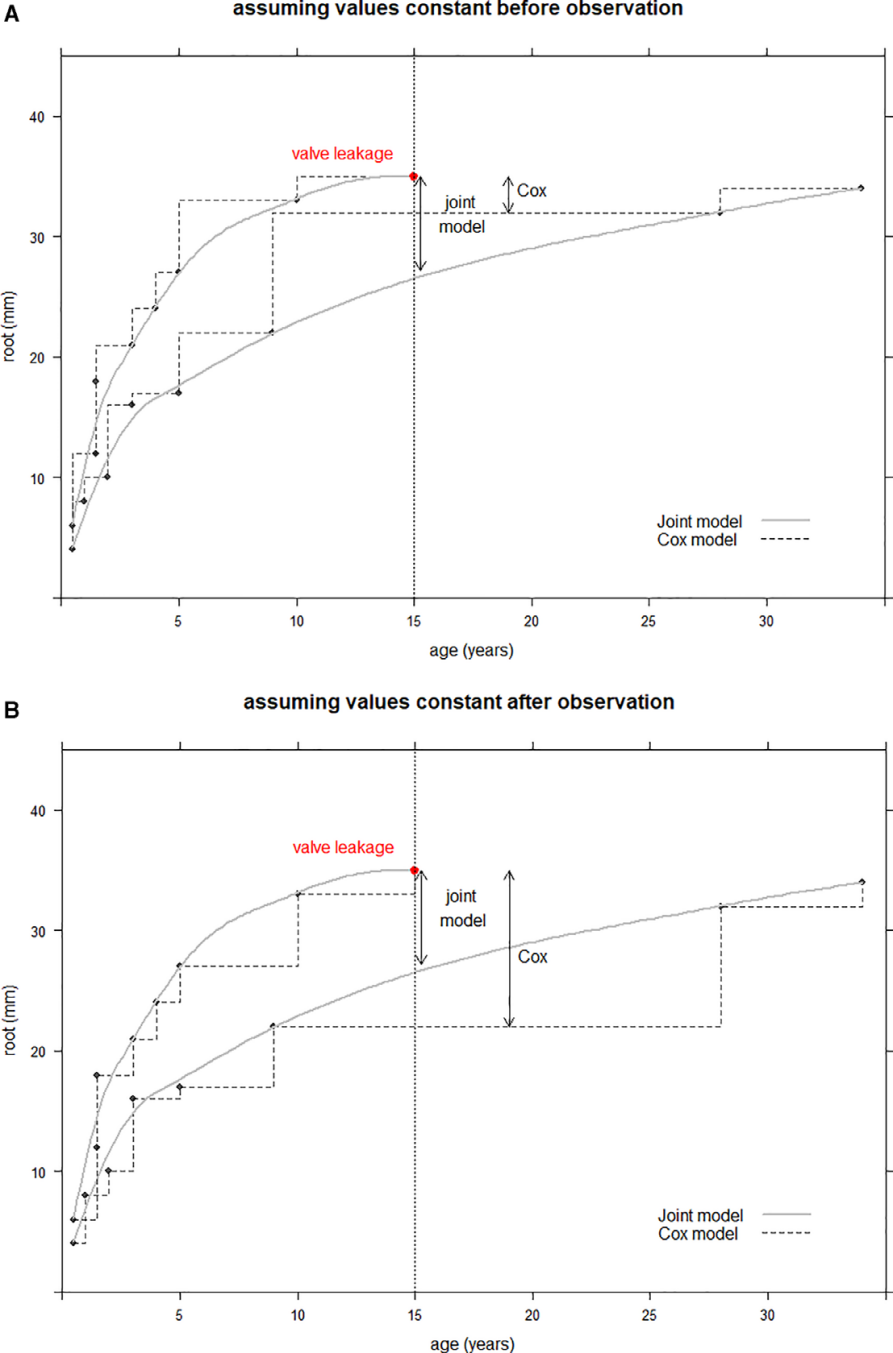
**Graphical representation of 2 versions of the time-dependent Cox model and the joint model (JM).** A graphical representation of 2 versions of the time-dependent version of the Cox model (TD Cox model) and the JM. The dots represent neoaortic root measurements of 2 fictional patients, one that reaches the event at 15 y and one that remains event-free. The dashed lines represent the values used by the TD Cox model in the estimation of the hazard, as a step-function path, and the solid line the values of the JM, following a more natural trajectory. In (**A**), the TD Cox model assumes the value to remain constant before the observation and in (**B**) after the observation. The 2-sided arrow represents for each model the value of the nonevent patient used in the hazard at the time of the event, showing a big difference in the 2 versions of the TD Cox model.

The aim of the analysis is to model time until certain degree of valve leakage (AR≥grade 2) is reached and to identify risk factors that contribute to the development of valve leakage, such as the echocardiographic measurements. One strategy that is traditionally used for analyzing time-to-event data, is a Cox proportional hazards model.^3^ When a risk factor is measured repeatedly over time, the Cox model can be extended to a time-dependent Cox model (TD Cox model), where the values of the longitudinal factor can change over time.^4^ A difficulty in such markers is that their values are only known at the visit times. Therefore, an assumption has to be made about their values in between visit times. The TD Cox model creates a step-function path of the time-dependent covariate and assumes that the value of the covariate stays constant in between 2 measurements when comparing marker values of patients with and without valve leakage. For this step function, it is possible to keep the value constant before or after the measurement. These options are visualized by the dashed lines in Figure 2A and Figure 2B, respectively.

The results of the TD Cox model can be found in Table 2 (TD Cox) and show the association between the repeatedly measured Root diameter and time to AR. The model additionally includes morphological subtype, sex, pulmonary valve morphology, previous pulmonary artery banding, and age at surgery >6 months as baseline covariates. Root diameter shows a nonsignificant association with the risk of AR, with a hazard ratio (HR) of 1.04 for each millimeter higher Root dimension (95% confidence interval [CI], 0.99–1.09). In this analysis, the value of the Root dimension was assumed constant in the time period before it was measured.

**Table 2.**
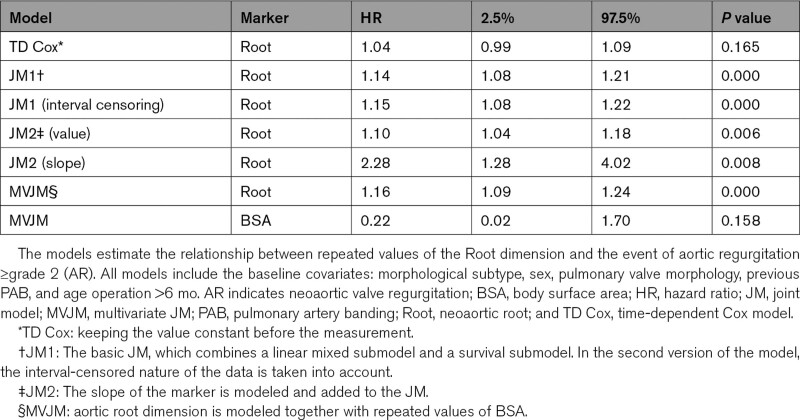
Results From the TD Cox and JMs

In creating a step-function path of the longitudinal outcome and using the measured value of the marker to estimate the hazard, the TD Cox model also implies there is no measurement error. In case of biomarkers, this representation of the longitudinal outcome usually does not correspond to the real world. In the data studied in this tutorial, the Root diameter will, most likely, grow gradually over time and will not stay constant between 2 measurements. When measurements are further apart in time, the TD Cox model will make a greater error, than if the measurements are close together in time. Additionally, the diameter needs to be assessed from an echocardiographic image, which can result in some measurement error. An additional assumption of the TD Cox model is that the availability of the measurement is not related to the event status. This indicates that the longitudinal marker needs to be an exogenous or external variable. Examples of exogenous variables could be the nurse taking the examination on a visit or the air quality in the area. The root diameter is a measurement taken from the patient and is, therefore, an endogenous or internal variable. Because of these simplifications and assumptions of the longitudinal outcome, the TD Cox model is not the most suitable model for analyzing these data.

### Joint Models

A more appropriate model to analyze data with these features is the JM for longitudinal and time-to-event data.^5–7^ The JM uses a separate regression model to describe the evolution of the marker over time and uses these estimated evolutions in a time-to-event relative risk model for the event of interest. A mixed-effects model is used to analyze the longitudinal marker over time, which combines fixed and random effects.^8,9^ The fixed effects estimate the population or average trajectory of the marker. In our example, the fixed effects model the average root diameter trajectory during follow-up. The random effects represent the deviation of each patient from this average trajectory. The random effects account for the correlation between repeated measurements from the same patient.

Instead of assuming a constant level of the longitudinal marker between observed values, the mixed-effects model results in an estimated value of the marker at each point in time. In the JM, this estimated evolution is related to the event status (ie, the estimated Root diameter at the time of the event is used for the relative risk analysis). This can be seen in Figure 2A and 2B, where the solid lines represent the estimated marker trajectories by the JM.

## Basic JM

To estimate the JM on the TGA patient data, first submodels for the longitudinal outcome and the survival outcome need to be specified.

For the longitudinal outcome, a mixed-effects model, as discussed in the previous section, is made to obtain an appropriate estimate of the evolution of the Root dilation. Based on Figure 1, we have seen that patients’ growth of the Root over time is not linear. This can be accounted for by specifying nonlinear time effects in the mixed model. There are multiple ways to model nonlinear effects in a model. Quadratic or higher-order polynomial terms can be added to the model to capture curvatures in the data. In this way the nonlinearity is modeled globally, meaning that one curve is fitted for the entire range of values of the covariate. There are scenarios where this will not provide an accurate fit of the data. For example, an upward curve could be shown for low values of the covariate, but a flat plateau phase for higher covariate values. In this case, we want to model the nonlinearity locally, which can be done using splines.^10,11^ With splines, the range of values of the covariate is broken up into pieces, and curves are fitted locally to the separate pieces. The breakpoints where the function changes are called knots. The number of knots and the location of the knots should be chosen by the researcher beforehand. Restricted cubic splines are a type of splines, fitted in such a way that the different functions are connected smoothly at the breakpoints. In the current data set, we used restricted cubic splines with 2 knots placed at 2 and 10 years postoperatively.^10,11^ The cubic splines are used in the fixed and random effects parts of the model.

Second, the survival submodel is specified, which is a relative risk model, similar to a Cox proportional hazards model. The longitudinal model for the root values is combined with the survival model by estimating the 2 models jointly. Now, instead of using the measured value of the marker in the survival submodel, the marker value estimated by the mixed-effects submodel is used (represented by the solid gray line in Figure 2).

Statistical software has been developed for estimating JMs, such as the JMbayes package in R.^12,13^ Details on evaluating the diagnostics of the JM can be found in previous publications (Rizopoulos, 2012^6^; Rizopoulos, 2016^13^). The results from the estimated JM (JM1) are shown in Table 2. The same baseline variables as in the TD Cox model are added as baseline covariates in the JM. Compared with the TD Cox model, the Root dimension shows a stronger and statistically significant association (HR, 1.14 [95% CI, 1.08–1.21]) with the event of AR (*P*<0.001).

### Extensions of the JM

In the TGA patient data, the event status (whether or not the patient has reached AR) is only obtained at the visit dates. This means that the event times are interval censored; the event probably did not take place exactly on the day the patient has a visit but rather somewhere between this day and the previous visit (when the patient was still without the event). This can be incorporated by estimating a survival submodel that takes interval censoring into account and is implemented in the software for the JM. The survival model that accounts for interval-censored data is a parametric survival model; here, we assumed a Weibull distribution for the event times. We reestimated the JM including the interval censoring information (JM 1–interval censoring) in Table 2 and the HR for the Root dimension showed very little difference between the 2 models (1.15 [95% CI, 1.08–1.22] versus 1.14 [95% CI, 1.08–1.21]).

### Different Association Structures

Apart from relating the value of the marker to the risk of the event, the joint modeling framework allows for extensions assessing additional associations. Perhaps it is not (only) the value of the marker that is related to the event but the fact that the marker is growing rapidly at that moment. The growth rate, that is, the slope of the marker, can be added to the JM to analyze this relationship. The results for the JM that uses both the value of the Root dimension and the growth rate, along with the same baseline covariates as earlier, are shown in Table 2 (JM2). This model is estimated with the survival submodel that accounts for interval censoring. The growth rate of the Root has a significant positive relation with the hazard of the event. This means that the faster the root increases, the higher the risk of valve leakage becomes (HR, 2.28 [95% CI, 1.28–4.02], *P*=0.008). In this model, the value of the root remains significant, so we can conclude that the velocity of the Root has additional information on top of its absolute value at each point in time. Incorporating neoaortic growth rate together with absolute neoaortic dimensions may give additional information on the risk of neoaortic regurgitation. In patients with aortopathy, current guidelines for surgical interventions on aneurysmatic ascending aortas are mainly based on absolute diameter thresholds. Lower aortic diameters thresholds can be considered in case of additional risk factors, of which rapid aortic growth rate or the presence of moderate-to-severe aortic valve insufficiency are some of these factors.

Apart from the slope of the longitudinal marker, multiple other features can easily be included in the JM: lagged effects (if there is a delay in timing between the marker and its effect on the event) and area under the curve (if the cumulative burden of a marker has an effect on the event), etc.

### Multiple Markers

The JM can also be extended by using multiple markers in the same JM. To illustrate this in our data, we included repeated values of the BSA of the patient in the model together with the Root dimensions. In this way, the association between the root diameter and the AR is conditioned on the BSA of the patient. This is of major importance in growing children, reflected by the TGA patient population used as example, but also serves for adult patients with different body stature. To estimate this multimarker model, a multivariate version of the mixed model is constructed, where the evolutions for both the root and the BSA are obtained. The multivariate mixed model additionally models the correlation between the 2 markers. The multivariate mixed model is combined with the survival model to obtain a multivariate JM. The multivariate JM is again estimated accounting for interval censoring.

In the multivariate JM, the estimated value of the Root appears to still have a significant association when corrected for repeated values of the BSA and the baseline covariates (HR, 1.16 [95% CI, 1.09–1.24], *P*<0.001), as seen in Table 2 (multivariate JM).

### Dynamic Predictions

**Figure 3.**
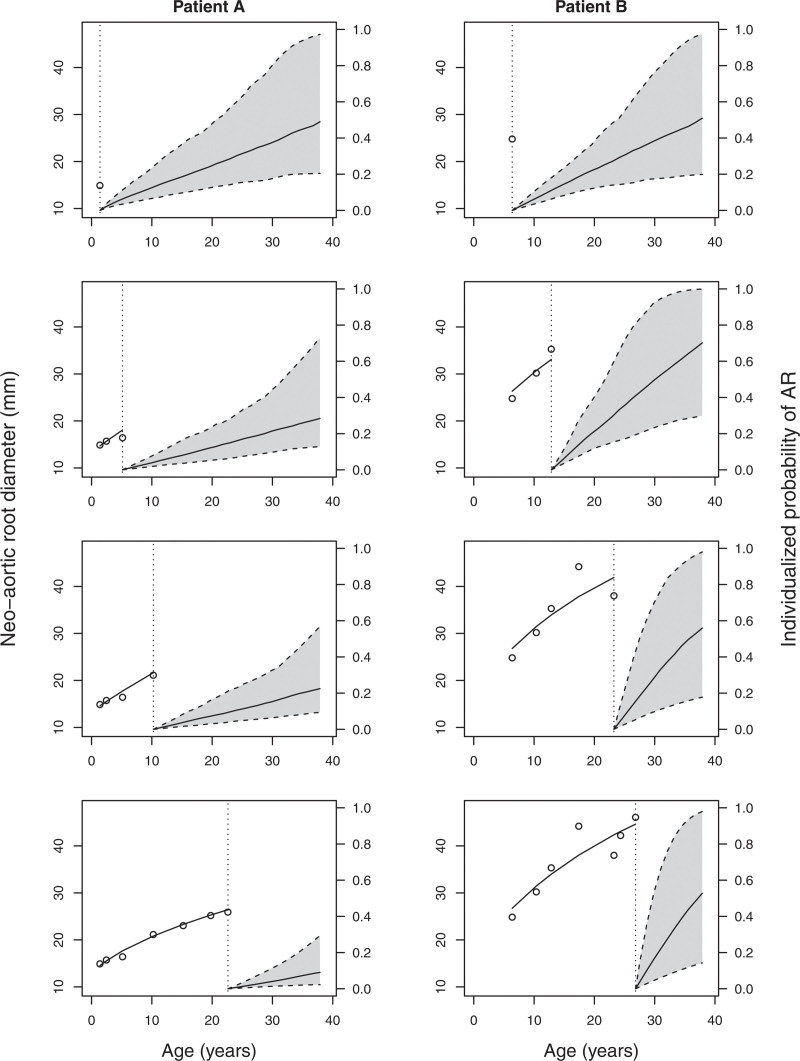
**Dynamic predictions of the incidence probability for 2 patients.** Dynamic predictions for 2 real patients. The **left** part on each graph shows the values of the neoaortic root diameter and the estimated trajectory by the joint model. The **right** part shows the estimated probability of the event for that patient, including the 95% CI. In each graph, new measurements are added and the probabilities are updated. AR indicates neoaortic valve regurgitation.

The estimated JM can be used to make individualized predictions. Based on a set of repeated values of the marker and relevant baseline covariates, the model can make predictions on future levels of the marker, and, more interestingly, on the probabilities of a future event.^14^ With graphs, it can be shown directly how adding new marker information updates the event probability of a patient. This is demonstrated in Figure 3 for 2 patients of the TGA study. Each patient has 4 plots, with an increasing number of root measurements. On the left part of each plot, the root values are plotted with the estimated trajectory through them. The right part of each plot shows the corresponding predicted event probabilities for that patient, which are equal to one minus the survival probabilities. The shaded gray area represents the 95% CI. Both patients are female with a TGA and intact ventricular septum diagnosis.

The 2 patients start with one measurement for the Root and have at that moment similar estimated event probabilities. After this, patient A shows limited growth of the root values that stabilizes over time. This is translated to lower event probabilities when more measurements are added to the prediction. For patient B, however, the root values grow more rapidly, with corresponding high event probabilities.

In addition, the plots can indicate whether there are any strong misspecifications in the fitted longitudinal trajectory. For both patients, the estimated longitudinal trajectory corresponds to the observed Root measurements.

The time-dependent Cox model, as discussed earlier in the tutorial, cannot be used to make survival predictions in a dynamic matter because the anticipated changes in future values of the time-varying covariate are not incorporated in calculating survival predictions for a single Cox model. A related method that could be used for dynamic predictions is a landmark approach. With the landmarking approach, multiple Cox models are estimated at different time points during follow-up, including only the patients that are still at risk set at the time of interest. The landmarking approach suffers from the same problem the time-dependent Cox model has in terms of dealing with endogenous covariates as discussed earlier in this tutorial. Previous studies have compared dynamic predictions in the landmarking and the JM approach, in real-life data sets and different simulations scenarios.^15–17^ In most scenarios, predictions based on the JM approach were better than the results from the landmarking approach; however, it might perform worse when the trajectory of the longitudinal marker is strongly misspecified in the JM (Rizopoulos et al^16^ and Ferrer et al^17^). This finding highlights the importance to evaluate the fit of your estimated model.

## Discussion

Studies with repeated marker measurements over time and a long follow-up provide us with a wealth of information, both on the trajectory of the marker and the risk of an event of interest. Currently, a time-dependent Cox model is often used to analyze the association between longitudinal measurements and a time-to-event outcome. For example, the data of this patient cohort after surgical repair for TGA was previously analyzed and reported in that way.^2^ However, this model is not optimal due to several restrictive assumptions that rarely hold in these types of data. Among them, the model keeps the marker constant between 2 time points of measurement. A JM is a more suitable option: it combines a mixed model with a relative risk survival model and relates an estimated value of the biomarker at each point in time to the risk of the event. Additionally, the JM allows for several extensions by incorporating other associations structures or combining multiple markers in the model. Moreover, the JM can be used to estimate event probabilities of patients in a dynamic manner, by updating these each time a new biomarker measurement is added.

In the illustrative example used in this article, repeated measurements of neoaortic dimensions are analyzed together with time to AR≥grade 2, in patients who underwent ASO for TGA with data from childhood to adulthood. Using a simpler time-dependent Cox model, the dimension of the Root does not appear to have a large nor significant association with AR. The JM, however, shows a different result. In this modeling approach, the Root shows a stronger and significant association with AR≥grade 2. These results from these additional JM analyses were reported recently.^18^ The JM additionally allows for inclusion of different aspects of a marker in the model. As shown, apart from using the diameter of the Root, the growth of these markers can be included in the model. In a different extension of the JM, a multimarker model can be constructed, illustrated by including the BSA which is of major importance in growing children with large changes in body stature over time. The use of dynamic predictions that can be obtained from the JM was illustrated using 2 patients selected from the TGA patient data. Based on their repeated values of the Root, event probabilities were estimated in a dynamic manner. This entails that each time a new measurement is obtained, the new information is incorporated in the estimated probability. The 2 patients showed different evolutions of their Root, which corresponded to different event probabilities. These dynamic predictions are implemented in software and can be made available to clinicians to update the event probabilities of a patient during each outpatient visit. Because the model incorporates the whole longitudinal trajectory, it is expected that these predictions based on the JM are more accurate than from more simple methods.

One major difference between the TD Cox model and the JM is how the models deal with the longitudinal marker at time points in between visits. As discussed, TD Cox keeps the value of the marker constant between 2 measurements, whereas the JM aims to estimate the complete trajectory. In the TGA patient data, these differences have led to contrasting conclusions on the association of the marker. In the TD Cox model we kept the value of the marker constant before the measurement as demonstrated in Figure 2A, which means that, in a sense, the model used information from the future. The value of the marker can also be assumed constant after the measurement instead of before, in which case a different value of the marker will be used in the TD Cox model (Figure 2B). In this version of the model, the HR of the TD Cox increased to 1.36 (95% CI, 1.26–1.46, *P*<0.001). This indicates that in this specific data set the TD Cox model is very sensitive to the choice of when to keep the marker constant, probably aggravated by the interval-censored nature of the data. This issue is not present in the JM (Figure 2A and 2B), indicating that the JM has a more robust way of dealing with the longitudinal outcome.

This JM methodology has important research advances in congenital and acquired cardiovascular disease, useful for future personalized decision making and predictive cardiovascular medicine as shown in this tutorial. Repeated imaging, laboratory, and clinical parameters over a period of time can be evaluated simultaneously for their impact on morbidity and mortality.

In congenital cardiology, for example, joint modeling can aid in optimizing the timing for surgical pulmonary valve insertion in repaired tetralogy of Fallot patients which is of ongoing debate. These patients suffer from chronic pulmonary regurgitation resulting in right ventricular volume overload with chamber dilatation (cardiac dimension, function, and heart failure laboratory values as longitudinal markers) and are at risk for the events cardiac dysfunction, arrhythmias, and sudden death (outcome parameters). Benefits of pulmonary valve implantation include recovery of cardiac function and chamber dilatation and joint modeling can aid in personalized decision making. Furthermore, joint modeling can serve as individualized prediction model to help prevent acute cardiac events (readmission, ventricular arrhythmias, or death) in patients treated for heart failure with echocardiographic, clinical, and laboratory measures as longitudinal markers for monitoring.

## Conclusions

Multiple modeling strategies are available to model the relationship between a longitudinal and a time-to-event outcome. Currently, a time-dependent version of the Cox model is used often; however, this model puts restrictive assumptions on the longitudinal marker. A more robust model is the JM for longitudinal and time-to-event data, which models the trajectory of the longitudinal outcome and relates this to the event of interest. Additionally, different associations and multiple markers can be incorporated into the JM, resulting in a flexible framework. The JMbayes package in R has been developed to easily run these models in practice. The software additionally allows calculating dynamic predictions of the survival outcome, so that the developed models can be used by clinicians treating patients.

## Article Information

### Sources of Funding

None.

### Disclosures

None.

### Supplemental Materials

R code

## Supplementary Material



## References

[1] Warnes CA Liberthson R Danielson GK Dore A Harris L Hoffman JI Somerville J Williams RG Webb GD. Task force 1: the changing profile of congenital heart disease in adult life. J Am Coll Cardiol 2001 37 1170 1175 doi: 10.1016/s0735-1097(01)01272-4 11300418 10.1016/s0735-1097(01)01272-4

[2] van der Palen RLF van der Bom T Dekker A Tsonaka R van Geloven N Kuipers IM Konings TC Rammeloo LAJ Ten Harkel ADJ Jongbloed MRM. Progression of aortic root dilatation and aortic valve regurgitation after the arterial switch operation. Heart 2019 105 1732 1740 doi: 10.1136/heartjnl-2019-315157 31292191 10.1136/heartjnl-2019-315157 PMC6855793

[3] Cox DR. Regression models and life-tables. J R Stat Soc B Stat Meth 1972 34 187 202 doi: 10.1111/j.2517-6161.1972.tb00899.x

[4] Therneau TM Grambsch PM. Modeling Survival Data: Extending the Cox Model 2000

[5] Tsiatis AA Davidian M. Joint modeling of longitudinal and time-to-event data: An overview. Stat Sinica 2004 14 809 834

[6] Rizopoulos D. Joint Models for Longitudinal and Time-to-Event Data: with Applications in R. Boca Raton, FL: CRC Press; 2012

[7] Wulfsohn MS Tsiatis AA. A joint model for survival and longitudinal data measured with error. Biometrics 1997 53 330 339 9147598

[8] Laird NM Ware JH. Random-effects models for longitudinal data. Biometrics 1982 38 963 974 doi: 10.2307/2529876 7168798

[9] Verbeke G Molenberghs G. Linear Mixed Models for Longitudinal Data 1997 Springer-Verlag

[10] Stone CJ Koo C. Additive Splines in Statistics. 1985 Proceedings of the American Statistical Association 45 48

[11] Harrell FE. Regression modeling strategies 2015 Springer

[12] R Core Team (2021) R: *A language and environment for statistical computing* Vienna, Austria R Foundation for Statistical Computing https://www.R-project.org/

[13] Rizopoulos D. The R package JMbayes for fitting joint models for longitudinal and time-to-event data using MCMC. J Stat Softw 2016 72 1 46

[14] Rizopoulos D. Dynamic predictions and prospective accuracy in joint models for longitudinal and time-to-event data. Biometrics 2011 67 819 829 doi: 10.1111/j.1541-0420.2010.01546.x 21306352 10.1111/j.1541-0420.2010.01546.x

[15] Suresh K Taylor JMG Spratt DE Daignault S Tsodikov A. Comparison of joint modeling and landmarking for dynamic prediction under an illness-death model. Biom J 2017 59 1277 1300 doi: 10.1002/bimj.201600235 28508545 10.1002/bimj.201600235 PMC5957493

[16] Rizopoulos D Molenberghs G Lesaffre EMEH. Dynamic predictions with time-dependent covariates in survival analysis using joint modeling and landmarking. Biom J 2017 59 1261 1276 doi: 10.1002/bimj.201600238 28792080 10.1002/bimj.201600238

[17] Ferrer L Putter H Proust-Lima C. Individual dynamic predictions using landmarking and joint modelling: validation of estimators and robustness assessment. Stat Methods Med Res 2019 28 3649 3666 doi: 10.1177/0962280218811837 30463497 10.1177/0962280218811837

[18] van der Palen RL Baart SJ van Geloven N Hazekamp MG Blom NA. Neoaortic growth rate and diameter as risk factors for neoaortic valve regurgitation after arterial switch operation. Heart 2020 106 1950 doi: 10.1136/heartjnl-2020-318142 33020229 10.1136/heartjnl-2020-318142

