# Supplementary material for: Joint Modeling of Longitudinal Markers and Time-to-Event Outcomes: An Application and Tutorial in Patients After Surgical Repair of Transposition of the Great Arteries
Source: Circ Cardiovasc Qual Outcomes. 2021 Oct 22;14(11):e007593. doi: 10.1161/CIRCOUTCOMES.120.007593 (PMC8598112; doi:10.1161/CIRCOUTCOMES.120.007593)
Supplement: Supplementary file 1 [file hcq-14-e007593-s001.pdf]

SUPPLEMENTAL MATERIAL – R CODE

```
###################################
##   Load necessary libraries    ##
###################################

library(JMbayes)
library(nlme)
library(latticeExtra)
library(splines)


###############################
##     Data preparation      ##
###############################

# Download the data from https://github.com/SaraBaart/Joint-Model-Tutorial
# and load into R
load("data.RData")

# The data set needs to be in the long format

# Variables in the data set:
# id    = Patient identifier
# y_a   = First longitudinal marker
# y_b   = Second longitudinal marker
# time  = Time of the longitudinal measurement
# time2 = Variable "time" shifted forward one measurement
#         Necessary for interval censored model
# Time  = Time of the event
# event = Event indicator (1 if event happened, 0 if censored)
# group = Binary covariate

# For the interval censored model, time cannot start at exactly 0
data$time <- ifelse(data$time == 0, data$time+0.01,
                    data$time)

# Make the data in the short format
data.id <-  data[!rev(duplicated(rev(data$id))),]

# Get the raw event rate for the  data set
table(data.id$event)[2] / nrow(data.id)

# Plot the data
xyplot(y_a ~ time | event, group=id, data = data, type = "l")



##############################################
##     Fit the time-dependent Cox model     ##
##############################################
##           Use y_a as biomarker           ##
##############################################


# TD-Cox model
TD.Cox <- (coxph((Surv(time, time2 , event) ~ y_a + group +
                    cluster(id)), data = data))

summary(TD.Cox)
```

```
########################################
##      Fit the basic joint model     ##
########################################
##        Use y_a as biomarker        ##
########################################

# Fit the survival model
Surv <- coxph(Surv(Time, event) ~ group,
                   data = data.id, x = TRUE, model = TRUE)

# Fit the mixed model
multMixedFit <- mvglmer(list(y_a ~ ns(time, knots = c(2,10)) + group  +
                               (ns(time, knots = c(2,10)) | id)),
                        data = data, families = list(gaussian))

# Fit the joint model
JM1 <- mvJointModelBayes(multMixedFit, Surv, timeVar = "time")

# Inspect the traceplots
plot(JM1)
# Obtain the results
summary(JM1)
# Obtain the HRs
exp(summary(JM1)$Survival)[,c(1,4,5)]



######################################################
##              Fit the basic joint model           ##
##  Accounting for interval censoring of the events ##
######################################################
##                Use y_a as biomarker              ##
######################################################

# Fit the survival model with IC data
SurvInt <- survreg(Surv(time, time2, event,  type = "interval") ~ group,
                   data = data.id, x = TRUE, model = TRUE)
summary(SurvInt)

# Fit the mixed model
multMixedFit <- mvglmer(list(y_a ~ ns(time, knots = c(2,10)) + group  +
                               (ns(time, knots = c(2,10)) | id)),
                        data = data, families = list(gaussian))

# Fit the joint model
JM1.IC <- mvJointModelBayes(multMixedFit, SurvInt, timeVar = "time")

# Inspect the traceplots
plot(JM1.IC)
# Obtain the results
summary(JM1.IC)
# Obtain the HRs
exp(summary(JM1.IC)$Survival)[,c(1,4,5)]
```

```
############################################
##            Fit the joint model         ##
##  Use slope as additional covariate     ##
############################################
##          Use y_a as biomarker          ##
############################################

# Define the associations: “value” and “slope”
Forms <- list("y_a" = "value",
              "y_a" = list(fixed = ~ 0 + dns(time, knots = c(2,10)),
                           indFixed = c(2:4) ,
                           random = ~ 0 + dns(time, knots = c(2,10)),
                           indRandom = 2:4, name = "slope"))

# Fit the second joint model
JM2 <- update(JM1, Formulas = Forms)

# Inspect the traceplots
plot(JM2)
# Obtain the results
summary(JM2)
# Obtain the HRs
exp(summary(JM2)$Survival)[,c(1,4,5)]



############################################
##          Fit the joint model           ##
##           Multimarker Model            ##
############################################
##           Use y_a and y_b              ##
############################################

# Fit the mixed model for two markers
multMixedFit2 <- mvglmer(list(y_a ~ ns(time, knots = c(2,10)) + group +
                                (ns(time, knots = c(2,10)) | id),
                              y_b ~ ns(time, knots = c(2,10)) + group +
                               (ns(time, knots = c(2,10)) | id)),
                         data = data, families = list(gaussian, gaussian))

# Fit the joint model
JM3 <- mvJointModelBayes(multMixedFit2, Surv, timeVar = "time")

# Inspect the traceplots
plot(JM3)
# Obtain the results
summary(JM3)
# Obtain the HRs
exp(summary(JM3)$Survival)[,c(1,4,5)]
```

```
##########################################
##      Make the dynamic predictions     ##
##########################################

# Make a data set for a specific patient A with the event
NDA <- data[data$id == 330,]

# Estimate survival probabilities for different time points
survPredsA <- vector("list", nrow(NDA))
for (i in 1:nrow(NDA)){
  survPredsA[[i]] <- survfitJM(JM1, newdata = NDA[1:i,], idVar = "id")
}

# Plot the graphs at four different time points
for (i in c(1,3,5,8)) {
  plot(survPredsA[[i]], ylab = "")
}


# Make a data set for a specific patient B without the event
NDB <- data[data$id == 253,]

# Estimate survival probabilities for different time points
survPredsB <- vector("list", nrow(NDB))
for (i in 1:nrow(NDB)){
  survPredsB[[i]] <- survfitJM(JM1, newdata = NDB[1:i,], idVar = "id")
}

# Plot the graphs at four different time points
for (i in c(1,3,5,7)) {
  plot(survPredsB[[i]], ylab = "")
}
```
